# Impact of Drug and Alcohol Use on Hospitalization for Injuries in Riders of Electric Bikes or Powered Scooters: A Retrospective Cross-Sectional Study

**DOI:** 10.3390/healthcare10061026

**Published:** 2022-06-01

**Authors:** Yafit Hamzani, Helena Demtriou, Adi Zelnik, Nir Cohen, Michael J. Drescher, Gavriel Chaushu, Bahaa Haj Yahya

**Affiliations:** 1 Department of Oral and Maxillofacial Surgery, Rabin Medical Center—Beilinson Hospital, Petach Tikva 4941492, Israel; drlilidemetriou@gmail.com (H.D.); gabi.chaushu@gmail.com (G.C.); 2 Maccabi-Dent, Holon 5810001, Israel; adi.zelnik@gmail.com; 3 Department of Orthopedic Surgery, Rabin Medical Center—Beilinson Hospital, Petach Tikva 4941492, Israel; nirco2@clalit.org.il; 4 Sackler Faculty of Medicine, Tel Aviv University, Tel Aviv 6997801, Israel; michaeldr@clalit.org.il; 5 Department of Emergency Medicine, Rabin Medical Center–Beilinson Hospital, Petach Tikva 4941492, Israel; 6 The Maurice and Gabriela Goldschleger School of Dental Medicine, Tel Aviv University, Tel Aviv 6997801, Israel; 7 Oral and Maxillofacial Private Clinic, Herzliya 4685107, Israel; bahaa.hag@gmail.com

**Keywords:** emergency department, alcohol, drugs, electric bikes, powered scooters, injury

## Abstract

The growing popularity of E-bikes and P-scooters has led to their increasing involvement in injuries. This study sought to evaluate the impact of drug and alcohol consumption on hospitalization rates for electric-vehicle-associated injuries. A retrospective cross-sectional study design was used, including patients evacuated to the emergency department (ED) of a tertiary medical center in 2014–2020 for injuries sustained while riding E-bikes or P-scooters. Data on clinical characteristics were collected from the medical files, including pre-accident usage of alcohol or drugs. Of the 1234 patients (75.7% male) who met the inclusion criteria, 90 (7.3%) were hospitalized. The mean (SD) number of admission days was 5.44 (±0.12). Alcohol consumption was associated with 2.2% of injuries and drug use with 0.6%. Patients who rode under the influence of alcohol were significantly more likely to be hospitalized than discharged (6.7% vs. 1.8%, χ^2^ (2) =19.25, *p* < 0.001); the odds ratio was 14.1. A similar association with hospitalization was found for drug use (χ^2^ (2) = 7.83, *p* = 0.02). Riding an E-bike or P-scooter under the influence of alcohol or drugs increases the probability of severe injury requiring hospital admission. These results should prompt the relevant authorities to initiate effective legislation of alcohol and drug use.

## 1. Introduction

Electric bicycles (E-bikes) and powered scooters (P-scooters) have become progressively widespread modes of transportation [1,2], and they are being increasingly recognized as a promising economical solution to urban last-mile delivery of people and goods. Their advantages include convenience, low cost, ease of use, and lower energy consumption compared to motorcycles and private cars. In most countries, no driving license is required [1,2,3,4]. Concerns have been raised, however, that the growing popularity of E-bikes and P-scooters has led to their increasing involvement in serious injuries [1,4]. The United States National Electronic Injury Surveillance System1 documented 133,872 E-bike- and P-scooter-associated injuries between 2007 and 2017, with E-bikes responsible for a high proportion of severe injuries requiring hospitalization relative to pedal bicycles. A recent study identified three main reasons for these findings: electromobility, driving under the influence of alcohol, and failure to wear a helmet (mainly on P-scooters) [5]. 

Overall, alcohol use is one of the leading risk factors for human death and disability, but its global association with health remains uncertain [6]. A routine screening survey in an emergency department (ED) found that alcohol-related E-bike and P-scooter injuries accounted for 13% of all presentations [7]. Others reported that nearly 18% of all reported traumas associated with E-scooters involved alcohol consumption [2].

E-bikes and P-scooters have become an important part of the urban environment, and the injuries associated with their use pose an increasingly large burden on patient health and healthcare. 

This study sought to evaluate the impact of drug and alcohol consumption on hospitalization rates for injuries sustained by E-bike and P-scooter riders.

## 2. Materials and Methods

A retrospective, cross-sectional study was conducted in the ED of a tertiary medical center from January 2014 to March 2020. The study protocol complied with the tenets of the Declaration of Helsinki and was approved by the local institutional ethics committee (approval number 0194-20-RMC). 

To select the cohort, a primary search of the electronic database of the ED was conducted using the following keywords: “electric scooter” and/or “electric bike” and/or “powered scooter” and/or “powered bike” and “injury/injured”. The medical files of the patients identified were further investigated to determine which were actually involved in an E-bike or P-scooter accident and had adequate data available for analysis. 

The following clinical parameters were collected from the medical files of the eligible patients: demographics, type of evacuation (self/ambulance/other), day and time of arrival to the ED, Glasgow coma scale (GCS) score on presentation, alcohol or drug use prior to the event, anatomic site(s) of injuries, need for hospitalization, and length of hospitalization. Findings were compared between patients who required hospitalization and patients who were discharged home from the ED. 

The data were analyzed using SPSS statistical software, version 25 (IBM^®^, Armonk, NY, USA). Continuous variables were summarized as means and standard deviations and discrete variables as frequencies. The chi-square test was used for univariate analysis of the relationship of each independent variable with the gravity of injury (hospitalization vs. discharge). Owing to the non-normal distribution of the continuous variables, the nonparametric Mann–Whitney test was used to compare independent samples. Univariate tests were done for all variables with association of hospitalization: gender, alcohol usage, drug usage, electric vehicle used, day and time of arrival to ED, evacuation method, patient injury (yes/no), face injury, head injury, and GCS upon arrival to ED. Significance was set at a *p*-value lower than 5%.

## 3. Results

### 3.1. Characteristics of Sudy Subjects

Of 1417 patients identified by the primary hospital database search, 1234 were found to have been involved in an E-bike or P-scooter accident on further investigation of the medical records and formed the final cohort. The full descriptive statistics are shown in Table 1. There were 934 men (75.7%) and 300 women (23.4%) of mean age 31.52 (±14.77) years. Ninety patients (7.3%) required hospitalization, and the remainder were discharged home from the ED. The mean number of admission days was 5.44 (±0.12). E-bikes were involved in 79.5% of the accidents and P-scooters in 20.5%. Alcohol consumption was involved in 2.2% of the injuries and drug use in 0.6%. Most patients arrived at the hospital on a weekday compared to the weekend (60.7% and 39.3%, respectively); 20.3% arrived during the morning hours (6 a.m. to 12 a.m.), 34.4% in the afternoon (12 a.m. to 6 p.m.), 31% in the evening (6 p.m. to 12 p.m.), and 14.3% at night (12 p.m. to 6 a.m.). Most patients (71.6%) self-evacuated to the hospital, and 19.9% were brought in by ambulance. Upper limbs were injured most frequently, in 55.8% of cases.

### 3.2. Main Results

Table 2 shows the distribution of the variables, and Table 3 shows the main results. Univariate analysis yielded a statistically significant association of alcohol-related injury with hospitalization (χ^2^ (2) = 19.25, *p* < 0.00). Patients with an alcohol-related injury were more likely to be hospitalized (6.7%) than discharged home from the ED (1.8%). On logistic regression analysis, the independent variables significantly predicted hospitalization (c^2^ (14) = 193.48, *p* < 0.001), explaining about 61.6% of the total variance. The model had an acceptable fit to the data (c^2^ (8) = 16.92, *p* = 0.03), classifying about 97.3% of the total observations. The odds ratio of an alcohol related-injury resulting in hospitalization was 14.1 relative to an injury that was not related to alcohol consumption (*p* < 0.001). The relationship between drug-related injury and hospitalization was also statistically significant (χ^2^ (2) = 7.83, *p* = 0.02).

Injuries related to drug use were more likely to require hospitalization (1.1%) than not (0.5%). The mode of evacuation was significantly related to hospitalization (χ^2^ (2) = 12.86, *p* = 0.002), with patients who were evacuated by ambulance being more likely to be hospitalized (26.7%) than discharged home (19.3%), and patients who self-evacuated being less likely to be hospitalized (56.7%) than discharged home (72.8%). Patients who were hospitalized had a significantly lower GCS score than those who were not hospitalized (14.81 ± 0.83 vs. 14.96 ± 0.65, *p* < 0.001).

### 3.3. Limitations of the Study

The current study design was retrospective and prospective and can aid in collecting wider and more detailed data regarding patients and injury characteristics, which may amplify significant statistic connections. Moreover, the study data were collected from ED referral of one medical center, and patients referred to this center most likely have similar norm habits and follow the same transport laws. Future studies may collect data from various medical centers, which could present global results and general recommendation for the riders population as well for the medical crews taking care of those riders following injuries associated with E-bikes or P-scooters.

## 4. Discussion

We investigated whether use of alcohol and drugs by riders of E-bikes and P-scooters impacted the severity of injuries they sustained in accidences involving these vehicles as measured by the likelihood of hospitalization compared to discharge from the ED.

Analysis of the demographic characteristics of our cohort yielded a male predominance, in agreement with the literature. However, men accounted for 75.7% of our cohort compared to only 50–62% in other studies [2,7,8,9,10,11]. The difference can be explained by the different study populations. For example, Mitchell et al. [11] (51.9% males) evaluated patients referred to three medical centers, one of which was a women’s emergency center, which may have skewed the results toward a higher proportion of female patients [11]. The mean age of the patients injured while riding E-bikes or P-scooters in our study was 31.5 years, which is similar to the 31.8–34 years reported in earlier diverse studies [2,8,9,10]. Thus, educational and awareness campaigns regarding injuries associated with E-bikes and P-scooters should be directed to this specific age group. 

In the present study, 19.9% of patients were evacuated to the ED by ambulance. Corresponding rates in similar studies varied widely from 7% to 44% [7,8,11]. Our finding that patients evacuated by ambulance were significantly more likely to be hospitalized (*p* = 0.002) may suggest proper decision making of the ambulance medical crew. This is supported by the significantly lower GCS score of the patients who were hospitalized compared to those who were not (*p* < 0.001).

Globally, alcohol consumption was shown to be the seventh leading risk factor for both deaths and disability-adjusted life years in 2016 [6], and the prevalence appears to be rising worldwide [6]. In our study, 2.2% of the cohort (90 of 1234 patients) reported consuming alcohol prior to the E-bike or P-scooter accident. This rate is considerably lower than that reported in similarly designed studies, ranging from 4.8% up to 28% [2,7,8,9,10,11]. The difference may be attributable to the different social norms of the populations and the smaller sample size in the previous studies (from 50 to 400) [2,7,8,9,10,11]. Moreover, the various studies used different parameters to assess alcohol consumption, such as documented intoxication, blood alcohol level, physician evaluation, and patient report [2,7,8,9,10,11]. We used mainly patient reports and medical crew clinical impression, which are inaccurate (often because of fear of law enforcement) and may have led to an underestimation. Heterogeneity of the sample may be another cause given that in the majority of studies, patients were referred to a specific medical center or geographically adjacent medical centers. 

In the two-month study of Mitchell et al. [11], 28% of 54 patients admitted consuming alcohol prior to the P-scooter accident. Nevertheless, alcohol consumption was not found to contribute to an increase in the incidence of hospital admissions (OR 1.25, 95% CI 0.17–9.01, Z = 0.22, *p* = 0.83) or operative management (OR 2.14, 95% CI 0.34–13.42, Z = 0.81, *p* = 0.42) [11]. By contrast, we found a significant relationship between alcohol-associated injury and hospitalization (*p* < 0.001), with riders who had consumed alcohol being 14.1 times more likely to require hospitalization (*p* < 0.001). The difference between our study and that of Mitchell et al. [11] may be attributable to differences in geographic location, cultural and social habits, sample size, and parameters used to measure alcohol consumption.

Unlike alcohol, the impact of drug and psychoactive substance use on E-bike and P-scooter injuries has hardly been investigated [2,5,7,8,12]. In the present study, a significant relationship was found between use of drugs prior to the accident and injury requiring hospitalization (χ^2^ (2) = 7.83, *p* = 0.02). 

Our results suggest that more stringent control policies and regulations for alcohol and drug use in E-bike and P-scooter riders may be needed in order to lower the severity of injuries and the rate of hospital admissions for those injuries. These findings should be corroborated by larger cooperative studies among medical centers worldwide.

## 5. Conclusions

We evaluated the severity of injuries associated with E-bikes and P-scooters and the impact of different variables on hospital admission. The increasing use of E-bikes and P-scooters has not been matched by adequate awareness of the hazardous impact of alcohol and drugs use in riders. The results showed that riding E-bikes and P-scooters under the influence of alcohol and drugs increases the probability of severe injury leading to hospitalization. The study may provide some guidance towards the formulation of effective safety legislation, law enforcement, and treatment programs, with special attention to the 30–40-year age group.

## Figures and Tables

**Table 1:** Demographic and clinical characteristics of injured E-bike and P-scooter riders.

Characteristic	*N*	Value
**Demographic characteristics**		
Gender		
Male	934	75.7%
Female	300	24.3%
Age (yrs.), mean ± SD		31.52 ± 14.77
**Injury characteristics**		
Alcohol-related		
No	958	77.6%
Yes	27	2.2%
Unknown	249	20.2%
Drug-related		
No	974	79.0%
Yes	7	0.6%
Unknown	252	20.4%
Vehicle		
P-scooter	253	20.5%
E-bike	980	79.5%
**Clinical characteristics**		
Time of arrival to ED		
Morning (6 a.m. to 12 a.m.)	250	20.3%
Noon (12 a.m. to 6 p.m.)	424	34.4%
Evening (6 p.m. to 12 p.m.)	383	31.0%
Night (12 p.m. to 6 a.m.)	177	14.3%
Day of arrival to ED		
Weekday	749	60.7%
Weekend	485	39.3%
Evacuation method		
Ambulance	245	19.9%
Self	884	71.6%
Other	105	8.5%
**Body site injured**		
Face	268	21.7%
Head	209	16.9%
Neck	31	2.5%
Chest	100	8.1%
Back	102	8.3%
Upper limbs	689	55.8%
Stomach and pelvis	118	9.6%
Lower limbs	609	49.35%
None	4	0.3%
**Hospital admission**		
No	1144	92.7%
Yes	90	7.9

ED, emergency department; values are *n* (%) unless otherwise indicated.

**Table 2:** Normal/non-normal distribution of study variables.

	Values	Normality Tests
**Gender**	Male/Female	
**Age**		Non-normal, *p* < 0.001
**Alcohol-related injury**	No/Yes/Unknown	
**Drug-related injury**	No/Yes/Unknown	
**Type of vehicle**	P-scooter/E-bike	
**Time of arrival to ED**	Morning (6 a.m. to 12 a.m.)/Noon (12 a.m. to 6 p.m.)/Evening (6 p.m. to 12 p.m.)/Night (12 p.m. to 6 a.m.)	
**Day of arrival to ED**	Weekday/Weekend	
**Evacuation method**	Ambulance/Self/Other	
**GCS**		Non-normal, *p* < 0.001
**Site of injury**	Face/Head/Neck/Chest/Back/Upper limbs/Stomach and pelvis/Lower limbs/None	
**Number of admission days**		Non-normal, *p* < 0.001

ED, emergency department; GCS, Glasgow coma scale.

**Table 3:** Univariate tests of association of all variables with hospitalization.

	Discharged	Hospitalized	
**Variable**	**Value**	**Value**	***P***
Gender			0.12
Male	856 (74.8%)	78 (86.7%)	
Female	288 (25.2%)	12 (13.3%)	
Alcohol-related			<0.001
No	902 (78.9%)	55 (61.1%)	
Yes	21 (1.8%)	6 (6.7%)	
Unknown	220 (19.2%)	29 (32.2%)	
Drug-related			0.02
No	913 (79.9%)	60 (67.4%)	
Yes	6 (0.5%)	1 (1.1%)	
Unknown	224 (19.6%)	28 (31.5%)	
Vehicle			0.34
P-Scooter	231 (20.2%)	22 (24.4%)	
E-bike	911 (79.8%)	68 (75.6%)	
Time of arrival to ED			0.07
Morning (6 a.m. to 12 a.m.)	234 (20.5%)	16 (17.8%)	
Noon (12 a.m. to 6 p.m.)	384 (33.6%)	40 (44.4%)	
Evening (6 p.m. to 12 p.m.)	355 (31.1%)	28 (31.1%)	
Night (12 p.m. to 6 a.m.)	170 (14.9%)	6 (6.7%)	
Day of arrival to ED			0.61
Weekday	692 (60.6%)	57 (63.3%)	
Weekend	450 (39.4%)	33 (36.7%)	
Evacuation			0.002
Ambulance	221 (19.3%)	24 (26.7%)	
Self	832 (72.8%)	51 (56.7%)	
Other	90 (7.9%)	15 (16.7%)	
Site of injury			
General No	8 (0.7%)	0 (0%)	0.43
Yes	1135 (99.3%)	90 (100%)	
Face No	785 (78.1%)	60 (81.1%)	0.55
Yes	220 (21.9%)	14 (18.9%)	
Head No	958 (84%)	72 (80%)	0.33
Yes	183 (16%)	18 (20%)	
GCS, mean ± SD	14.96 ± 0.65	14.81 ± 0.83	<0.001

ED, emergency department; GCS, Glasgow coma scale; all values are *n* (%) unless otherwise indicated.

## Data Availability

The datasets used and/or analyzed during the current study are available from the corresponding author on reasonable request.

## References

[1] DiMaggio C.J., Bukur M., Wall S.P., Frangos S.G., Wen A.Y. (2019). Injuries associated with electric-powered bikes and scooters: Analysis of US consumer product data. Inj. Prev..

[2] Trivedi B., Kesterke M.J., Bhattacharjee R., Weber W., Mynar K., Reddy L.V. (2019). Craniofacial injuries seen with the introduction of bicycle-share electric scooters in an urban setting. J. Oral Maxillofac. Surg..

[3] Gojanovic B., Welker J., Iglesias K., Daucourt C., Gremion G. (2011). Electric bicycles as a new active transportation modality to promote health. Med. Sci. Sports Exerc..

[4] Ishmael C.R., Hsiue P.P., Zoller S.D., Wang P., Hori K.R., Gatto J.D., Li R., Jeffcoat D.M., Johnson E.E., Bernthal N.M. (2020). An early look at operative orthopaedic injuries associated with electric scooter accidents: Bringing high-energy trauma to a wider audience. J. Bone Joint Surg. Am..

[5] Meyer H.L., Kauther M.D., Polan C., Abel B., Vogel C., Mester B., Burggraf M., Dudda M. (2022). E-scooter, e-bike and bicycle injuries in the same period-A prospective analysis of a level 1 trauma center. Unfallchirurg.

[6] GBD 2016 Alcohol Collaborators (2018). Alcohol use and burden for 195 countries and territories, 1990–2016: A systematic analysis for the Global Burden of Disease Study 2016. Lancet.

[7] Beck S., Barker L., Chan A., Stanbridge S. (2020). Emergency department impact following the introduction of an electric scooter sharing service. Emerg. Med. Australas..

[8] Badeau A., Carman C., Newman M., Steenblik J., Carlson M., Madsen T. (2019). Emergency department visits for electric scooter-related injuries after introduction of an urban rental program. Am. J. Emerg. Med..

[9] Bekhit M.N.Z., Le Fevre J., Bergin C.J. (2020). Regional healthcare costs and burden of injury associated with electric scooters. Injury.

[10] Trivedi T.K., Liu C., Antonio A.L.M., Wheaton N., Kreger V., Yap A., Schriger D., Elmore J.G. (2019). Injuries associated with standing electric scooter use. JAMA Netw. Open.

[11] Mitchell G., Tsao H., Randell T., Marks J., Mackay P. (2019). Impact of electric scooters to a tertiary emergency department: 8-week review after implementation of a scooter share scheme. Emerg. Med. Australas..

[12] Hamzani Y., Bar Hai D., Cohen N., Drescher M.J., Chaushu G., Yahya B.H. (2021). The impact of helmet use on oral and maxillofacial injuries associated with electric-powered bikes or powered scooter: A retrospective cross-sectional study. Head Face Med..

